# Digital financial inclusion and household financial vulnerability: An empirical analysis of rural and urban disparities in China

**DOI:** 10.1016/j.heliyon.2024.e35540

**Published:** 2024-07-31

**Authors:** Jianhe Liu, Yuan Chen, Xuanyu Chen, Bin Chen

**Affiliations:** School of Finance, Zhejiang University of Finance and Economics, China

**Keywords:** Household financial vulnerability, Digital financial inclusion, Financial vulnerability, Financial risk

## Abstract

In recent times, a notable increase in the leverage ratios among numerous households across China has been witnessed, culminating in heightened household financial vulnerability. Concurrently, the sphere of digital inclusive finance has witnessed rapid advancement, establishing itself as a crucial mechanism for Chinese households to counteract financial risk shocks. This research article meticulously constructs an ordered regression model, anchored in micro-level data from household surveys, to delve into the influence and operative mechanisms of digital inclusive finance on the vulnerability of household finances. Empirical findings from this study robustly indicate that the evolution of digital inclusive finance significantly mitigates the household financial vulnerability. A thorough mechanism analysis reveals that digital inclusive finance primarily curtails household financial vulnerability through several avenues: it notably enhances financial literacy, augments the income derived from household financial assets, and elevates contributions to commercial insurance. Intriguingly, a heterogeneity analysis underscores that the impact of digital inclusive finance is more pronounced in reducing financial vulnerability amongst households registered in rural areas and those with lower income levels. This article contributes to the expansion of the theoretical framework concerning household financial vulnerability, offering insightful guidance and policy implications for addressing financial vulnerability concerns and forestalling macro-financial risks.

## 1 Introduction

The realm of household financial risk management has perennially captivated academic interest, primarily focusing on the concept of financial vulnerability as a metric for assessing a household's susceptibility to financial risk. Despite the plethora of scholarly discourse, a consensus on a definitive characterization of household financial vulnerability remains elusive [1]. Prevailing academic thought often correlating financial vulnerability with factors such as household income and debt levels [2]. The prevailing rationale posits that financial vulnerability escalates in the face of income instability or excessive debt-to-income ratios. It has also been suggested that financial vulnerability is associated with the ratio of liabilities to assets [3,4]. O'Connor et al. [5] offer a different perspective, viewing financial vulnerability as the propensity of a household to encounter financial distress. This paper proposes a comprehensive definition of household financial vulnerability, positing it as a reflection of market risk at the household level. This encompasses both diversifiable risks, manageable through financial strategies, and inherent risks that are intractable. Therefore, this paper defines household financial vulnerability as the household resilience to adverse financi^1^al shocks.

The “China Leverage Ratio Report 2023,” issued by the National Institute for Financial Development, indicates that China's macro leverage ratio climbed to 287.8 %, with the residential sector's leverage ratio reaching 63.5 %. This is a substantial increase from 2008, when the overall macro leverage ratio was only 141.2 %, and the residential sector's ratio stood at 17.9 %. The macro leverage ratio measures the debt of non-financial sectors relative to the country's annual GDP, encompassing residents, non-financial enterprises, and the government. A higher ratio suggests an elevated overall debt burden. Over the 15-year span from 2008 to 2023, the leverage in China's real economic sectors has surged, primarily driven by the residential sector. This increase in leverage ratios has heightened the risk of debt defaults, thereby augmenting financial vulnerability across numerous households [6]. This phenomenon is partly attributable to limited avenues for mitigating household risk [7]. This is particularly acute among agricultural households, who face substantial challenges in accessing formal financial services compared to urban counterparts, thereby exacerbating their financial vulnerability.

The household economy, a critical component of the broader social economy, possesses the potential to propagate risk shocks from the micro to the macroeconomic level, potentially culminating in a financial crisis [8]. In response, the 20th Party Congress report underscores the imperative of diversifying income sources for middle and low-income groups and enhancing property income among urban and rural residents. It advocates for comprehensive rural revitalization, which includes bolstering agricultural support and improving rural financial services. The objective of this paper is to explore strategies for providing efficient and accessible services to groups, including agricultural households, to forestall financial risk shocks, mitigate household financial vulnerability, and ensure a harmonious interplay between the financial industry's robust operation and high-quality economic development.

Empirical studies indicate that household financial vulnerability is influenced by a constellation of factors. First, household characteristics significantly influence financial vulnerability. Research indicates that households with male heads are generally less financially vulnerable than those headed by females [9]. Regarding age, older household heads are often associated with less financial vulnerability compared to younger ones. This trend is attributed to the increased accumulation of wealth as household heads age, thereby reducing financial vulnerability [10]. Additionally, higher educational attainment among household heads correlates with increased assets and income, which help buffer financial shocks, thus reducing vulnerability [11]. Secondly, household economic factors play a crucial role in financial vulnerability. Commonly, wealthier households with substantial assets exhibit lower financial vulnerability [3,12]. However, some studies suggest that financial vulnerability can increase among middle- and high-income households as their income rises, potentially due to increased liabilities associated with income expansion [8]. An increase in liabilities, especially when coupled with rising home equity, also escalates financial vulnerability [13]. Moreover, unemployment markedly increases household financial vulnerability [14]. Thirdly, the influence of financial literacy on household financial vulnerability has been extensively studied [15]. Findings generally show a significant negative correlation between financial literacy and vulnerability [16]. Research during the COVID-19 pandemic across countries like Australia and France highlights that greater financial literacy and internal control significantly mitigate financial vulnerability [17]. It is imperative to acknowledge the burgeoning growth of digital financial inclusion in China in recent years, a development largely attributable to advancements in high technologies such as artificial intelligence, big data analytics, and cloud computing. As reported in the Analysis Report on China's Financial Inclusion Indicators (2021) by the People's Bank of China (2022), the number of mobile payment transactions processed by Chinese banking institutions has surpassed a hundred billion yuan, with a transaction amount reaching 2353.96 trillion yuan. Furthermore, the transaction funds of China's non-banking payment institutions have exceeded 355.46 trillion yuan, marking a year-on-year increase of 20.67 %.

The rapid development of digital inclusive finance has profoundly positive implications for both the economy and society. Firstly, it effectively mitigates the financing constraints typically imposed by traditional banking institutions, thereby broadening the financing avenues available to small and medium-sized enterprises (SMEs). This expansion in funding opportunities facilitates increased production scales for SMEs, fostering economic value creation [18]. Secondly, digital inclusive finance fosters innovation among high-tech enterprises [19] and drives technological advancements within SMEs [20]. The combination of low transaction costs and accessible financing conditions also stimulates entrepreneurial endeavors [21,22], ultimately bolstering economic growth [23]. Thirdly, digital finance addresses issues of geographic and price-based financial exclusion through the fusion of technology and finance [24,25]. Its cost-effectiveness, user-friendly interfaces, and widespread accessibility enable households to diversify their asset portfolios, thus enhancing the accumulation of financial assets and income growth [26]. Particularly in remote rural areas, where financial services are scarce and costly to access [27], digital inclusive finance alleviates the financial burdens of households [28], leading to increased income and improved living standards [29,30]. Moreover, it facilitates rational allocation of financial assets, enhances resource utilization efficiency, and optimizes investment strategies [31], consequently mitigating income inequality and reducing the wealth gap between socioeconomic strata [26].

The deeper integration of Internet technology with financial inclusion has significantly broadened investment channels and improved access to financial information for households [32]. As financial services become more readily available and household participation in financial markets increases, their capacity to manage risks is enhanced, consequently reducing financial vulnerability [33]. The poverty-reducing effects of digital investment and digital credit are especially pronounced in households with higher financial literacy [34]. Furthermore, the online transaction methods facilitated by digital payments effectively lower transaction costs in financial dealings and boost income from financial assets [35], with higher income levels correlating with reduced financial vulnerability [36]. Additionally, the availability of online insurance products addresses the limited ability of some households to handle risk shocks by providing essential risk protection and lessening the impact of such shocks [37,38]. From an employment and entrepreneurship perspective, digital finance significantly eases credit constraints for firms [39,40], which supports capacity expansion and increases labor demand [41,42], thereby creating employment opportunities that mitigate the adverse effects of employment shocks on household financial vulnerability [17]. For entrepreneurs, the availability of financial resources not only spurs business creation but also fosters job creation [43]. Moreover, increased employment boosts household income, further preventing households from slipping into poverty [44].

The existing literature on the role of digital financial inclusion in mitigating household financial vulnerability exhibits several gaps. Firstly, much of the research emphasizes traditional financial inclusion and overlooks digital financial inclusion [33,45,46], which, as a distinct facet of fintech, offers unique functionalities through digital technology that traditional methods cannot, thus having a more pronounced effect on reducing household vulnerability [44]. Secondly, while numerous studies explore the poverty reduction capabilities of digital inclusive finance, they often neglect its impact on financial vulnerability [34,47]. For example, some research highlights the significant reduction in the likelihood of households entering relative poverty due to digital inclusive finance, without addressing broader financial vulnerability aspects [47]. Thirdly, the literature frequently focuses on specific household demographics such as rural, urban, or elderly households [44,48]. Although these studies are targeted, there is a scarcity of research addressing the traits of financial vulnerability that are broadly applicable across various household types. Fourthly, the assessment of household financial vulnerability often categorizes it as low, medium, or high based solely on debt levels and emergency savings, thereby ignoring daily financial flow dynamics within households [36]. Lastly, while some analyses suggest that enhancing productivity, fostering entrepreneurship, and boosting employment are key mechanisms for reducing rural household vulnerability to poverty and highlighting the prevention of financial risks, the protective role of financial derivatives like insurance post-risk occurrence merits further exploration [48].

Addressing these identified gaps, this paper introduces an analysis of household financial vulnerability by incorporating the dimension of liquidity levels alongside traditional metrics such as emergency response capacity and indebtedness [36]. It examines how digital financial inclusion influences household financial vulnerability. In the mechanism analysis, the study identifies three intermediary channels—financial literacy, household asset income, and commercial insurance—considering their critical roles in household income, financial management strategies, and the risk transfer functions of commercial insurance. This approach provides a more comprehensive understanding of the factors that mitigate financial vulnerability through the lens of digital financial inclusion.

In this research, we employ the 2019 China Household Finance Survey (CHFS) data and the 2018 Digital Financial Inclusion Index of China (DFIIC) data, positioning digital financial inclusion as the pivotal explanatory variable. This study aims to empirically assess the influence of digital financial inclusion on the financial vulnerability of Chinese households. To ensure methodological rigor, we meticulously select the control variables at all levels affecting household financial vulnerability, address the potential endogeneity problem and conduct robustness tests. Employing a multifaceted approach, we dissect the direct and indirect mechanisms of impact, and subsequently, conduct a heterogeneity analysis for specific sample subgroups.

The findings of this study are significant: we ascertain that the progression of digital financial inclusion attenuates household financial vulnerability. More precisely, for each unit increment in the digital financial inclusion index, there is a corresponding decrease in household financial vulnerability by 1.9883 units. Furthermore, our analysis indicates that households with a head who is in sound health, possesses higher educational attainment, and is male, exhibit a reduced propensity towards financial distress. Conversely, households characterized by a larger size and a higher proportion of home equity are more susceptible to financial vulnerability. The mechanism analysis delineates three primary pathways through which digital financial inclusion impacts household financial vulnerability: enhancing the financial literacy of the household head, increasing income from household financial assets, and augmenting the purchase of household commercial insurance. Notably, the enhancement of the household head's financial literacy emerges as the most efficacious factor in reducing household financial vulnerability. In the realm of heterogeneity analysis, this paper discovers that the mitigating effect of digital financial inclusion is particularly pronounced among rural households and those in lower income brackets.

This research makes a substantive contribution to the existing body of theoretical studies on household financial vulnerability. It delves into the intricate dynamics between digital inclusive finance and the financial vulnerability of Chinese households, thereby offering a more nuanced and comprehensive theoretical framework. This study's contributions are threefold: firstly, it expands the research scope to encompass a wider array of household types, providing broad theoretical and practical insights; secondly, this paper reinterprets the concept of household financial vulnerability by categorizing households into four distinct levels of financial vulnerability, based on liquidity level, contingency capacity, and level of indebtedness. This classification provides a nuanced framework for understanding the varying degrees of financial risk faced by households; thirdly, this paper elucidates the transmission mechanisms of digital financial inclusion in mitigating household financial vulnerability, encompassing the enhancement of financial literacy, increase in household financial asset income, and promotion of commercial insurance purchase.

This research serves dual purposes: it assists households, including those with low income and agricultural backgrounds, in mitigating financial vulnerability through the adoption of digital financial inclusion products and services, thereby enhancing their resilience against financial shocks. Concurrently, it offers valuable insights to government and policy makers, providing a comprehensive micro and macro perspective on household financial vulnerability, which can be instrumental in policy formulation and adjustment.

## 2 Theoretical mechanisms and research hypotheses

This study delineates the multifaceted impact of digital financial inclusion on household financial vulnerability, focusing on three critical dimensions: liquidity level, contingency capacity, and household indebtedness.

### 2.1 Direct impact of digital financial inclusion on household financial vulnerability

In the contemporary landscape, China's financial sector is experiencing robust growth, catalyzed by technological advancements. This evolution has precipitated a diversification in the business and product offerings of financial institutions, giving rise to new entities within the financial ecosystem. Beyond traditional entities like commercial banks and insurance companies, the emergence of brokerage firms, fund companies, trust companies, and other financial institutions has significantly contributed to the multi-channel, multi-dimensional, and multi-level development of high-quality financial services [49]. A pivotal aspect of this transformation is the proliferation of online financial products and services, which have radically altered the investment and money management behaviors of residents, this family is also more likely to accumulate wealth [50].

The expansion of financial service channels, facilitated by digital inclusion, has democratized access to financial services, transcending geographical and temporal barriers. This accessibility has not only diversified the operations of financial institutions but also invigorated the financial engagement and investment zeal among individuals. Households, leveraging the spectrum of investment opportunities and financial services, including varied loan products afforded by digital financial inclusion, can enhance their liquidity, fortify their capacity to respond to emergencies, and judiciously manage their indebtedness, thereby ameliorating their financial vulnerability.

Given this backdrop, the following hypothesis is posited:

H1 The advancement of digital financial inclusion serves as a mitigating force against household financial vulnerability.

### 2.2 Indirect impact of digital financial inclusion on household financial vulnerability

Financial literacy emerges as a pivotal determinant influencing an investor's strategic approach to investment and financial management. When the principal decision-maker of a household exhibits low financial literacy, it often translates into a limited understanding of financial products and services. This deficit can manifest in two critical ways: firstly, the decision-maker might be disproportionately attracted to financial products offering high returns without adequately considering the associated risks, thereby escalating the household's financial risk. Secondly, a lack of awareness regarding potential risks may lead to a portfolio lacking in liquidity, ill-prepared to manage sudden debt crises or large expenses. This scenario frequently culminates in suboptimal decision-making, especially in the face of unanticipated risk shocks [51].

Digital financial inclusion plays a transformative role in the realm of investment decision-making by economizing the time and effort required for information gathering. Financial institutions, leveraging digital platforms, provide customers with pertinent advice online, thereby enriching their understanding of financial products and services. This process facilitates effective asset risk management and enhances the financial literacy of the population [52]. Households with heightened financial literacy typically demonstrate a more rational allocation of assets and a scientific approach to managing financial risks [53].

Digital inclusive finance subtly promotes the dissemination of diverse financial knowledge, thereby enhancing the financial literacy of household heads or economic decision-makers. This elevated financial literacy enables these individuals to formulate rational financial policies for their households, optimizing savings levels and debt proportions. This, in turn, enhances their capacity to respond to emergencies and manage indebtedness more effectively, fortifying the household's resilience against adverse risk shocks and consequently improving its financial vulnerability status.

In light of the above discussion, the following hypothesis is posited:

H2 Digital financial inclusion can ameliorate household financial vulnerability by elevating the financial literacy of household heads.

Income from household financial assets is defined as the additional revenue generated through the management of owned financial assets. The advent of digital inclusive finance has introduced a plethora of investment and financial management options, catering to a wide spectrum of investor risk preferences and thereby augmenting the financial asset income of residents [54]. In practical terms, families with substantial collateral assets or stable economic income are often prioritized in accessing financial services like loans, given their consistent cash flow and collateral security. These households, even in the face of unexpected risk shocks, possess adequate coping mechanisms and preventive strategies. Conversely, many low- and middle-income households face financial exclusion, hindering the growth of their financial asset income. Digital inclusive finance, with its foundational principles of being 'grassroots' and ‘inclusive’, broadens access to financial services for these households, enhancing their liquidity levels and reducing the likelihood of financial distress.

Thus, digital financial inclusion indirectly mitigates household financial vulnerability through the channel of increased household financial asset income. Accordingly, the following hypothesis is proposed:

H3 Digital financial inclusion can reduce household financial vulnerability by augmenting the income derived from household financial assets.

Regarding risk shock prevention, commercial insurance stands as a viable option for investors to manage risk. Distinguished from other assets that yield direct economic returns, insurance products primarily function to provide financial compensation in the event of natural disasters or accidents, based on pre-agreed contractual terms with the insurer, thereby mitigating property loss for customers. The proportion of insurance assets in a household's portfolio reflects the risk management strategies of household decision-makers, and an increase in such assets aids in enhancing the household sector's emergency response capacity through risk diversification [55,56]. Digital inclusive finance, with its burgeoning array of online financial products, including insurance offerings, has expanded the scope and accessibility of insurance products. Platforms like Alipay have played a significant role in promoting commercial insurance among residential households, implying an enhancement in the household sector's emergency response capacity. The presence of commercial insurance improves the household sector's flexibility in responding to risks and diminishes household financial vulnerability.

Therefore, the following hypothesis is posited:

H4 Digital financial inclusion facilitates an increase in the purchase of commercial insurance by resident households, thereby attenuating their financial vulnerability.

## 3 Research design and data sources

### 3.1 Data sources

This investigation utilizes data from the China Household Finance Survey (CHFS), administered by the China Household Finance Survey and Research Center at Southwestern University of Finance and Economics, in conjunction with the Digital Financial Inclusion Index (DFIIC) from Peking University.

#### 3.1.1 Explained variables

The explained variables are derived from the 2019 CHFS at the provincial household level. This paper incorporates three key indicators reflective of the dimensions of liquidity level, emergency capacity, and indebtedness: the income-expenditure gap, the level of emergency savings, and the level of debt. These indicators are operationalized as dummy variables, assigned values based on pre-established criteria, and subsequently summed up to construct an overall household financial vulnerability indicator.

Firstly, the income-expenditure gap indicator assesses whether a household's annual income surpasses its annual expenditure. This indicator is assigned a value of 0 in cases of a surplus and 1 in instances of a deficit.

Secondly, the emergency savings level indicator evaluates the adequacy of a household's savings in addressing unforeseen risks. This measure is designated as 0 if the household's savings (inclusive of bank demand deposits and cash) exceed three months' worth of daily expenses, and 1 if they fall below this threshold.

Lastly, the debt level indicator quantifies the household's annual debt as a proportion of its annual income. A ratio of 30 percent or less yields an indicator value of 0, whereas a higher ratio results in a value of 1.

The composite financial vulnerability indicator, ranging from 0 to 3, serves as a gauge of financial vulnerability, with higher values indicating greater vulnerability and a value of 0 signifying the absence of vulnerability. This nuanced approach to measuring financial vulnerability provides a comprehensive understanding of the multifaceted financial challenges faced by households.

#### 3.1.2 Explanatory variables

For the explanatory variables, this study employs the 2018 provincial Digital Financial Inclusion Index of China (DFIIC), lagged by one period, to mitigate potential endogeneity issues that could arise from the influence of explained variable on explanatory variables within the same period. The DFIIC values are logarithmically transformed to address potential non-linear relationships and to facilitate a more nuanced analysis.

#### 3.1.3 Control variables

Drawing from the 2019 CHFS data, the control variables are defined as follows:

- Nature of Household Registration: Assigned a value of 1 for agricultural registered permanent residence and 0 for others.
- Household Size: Quantified as the total number of individuals within the household.
- Age of Head of Household: The actual age of the household's primary decision-maker.
- Health Status of Head of Household: Categorized on a scale from high to low health status, with values ranging from 1 to 5.
- Educational Level of Head of Household: Assigned values from 1 to 9, indicating literacy level from low to high.
- Gender of Head of Household: Female heads are assigned a value of 1, and male heads a value of 0.
- Marital Status: The marital status of household members is categorized into six levels, with values ranging from 1 to 6.
- Social Capital of Household: Reflects the employment of household members in government institutions and organizations.
- Child Rearing Ratio: The proportion of children within the household.
- Elderly Dependency Ratio: The proportion of elderly individuals within the household.
- Real Estate: Calculated as the proportion of housing assets in the total assets.
- Gross Family Income from Agriculture: The annual gross family income from agriculture, logarithmically transformed.
- Gross Non-Agricultural Income: Total annual non-agricultural income of the household, logarithmically transformed.
- Household Net Worth: Annual total household assets minus total liabilities, logarithmically transformed.

#### 3.1.4 Mediating variables

Sourced from the 2019 CHFS, the mediating variables are as follows:

- Financial Literacy: Reflecting the household head's level of financial knowledge, this variable is derived from responses to survey questions about interest rates, inflation, and economic news, processed through principal component factor analysis.
- Household Income from Financial Assets: Calculated based on the profit and loss generated from all financial product transactions recorded in the questionnaire, using the logarithmic value of the profit and loss amount.
- Total Commercial Premiums: Representing the household's expenditure on commercial insurance, this variable is operationalized as the logarithmic value of the total annual commercial premiums paid by all household members.

### 3.2 Variable selection and modeling

The explained variable in this research is household financial vulnerability. Recognizing the inherent measurement errors often associated with the collection of household income data, this study uses the difference between income and expenditure as a proxy for it. However, beyond the mere level of income and expenditure, this paper argues that household debt and savings levels are equally pivotal in assessing financial vulnerability. Consequently, household financial vulnerability is disaggregated into three distinct dimensions for a more holistic analysis: liquidity level, indebtedness level, and contingency capacity.

The central explanatory variable in this study is derived from the Peking University Digital Financial Inclusion Index. In the context of robustness testing, this research utilizes three subsidiary indicators as alternative explanatory variables: the breadth of digital coverage, the depth of use, and the degree of digitization.

Concerning control variables, this study incorporates factors such as the nature of household registration, household size, the age of the household head, and the head's health status. These variables are instrumental in capturing the effects of divergent individual characteristics of household heads on their financial vulnerability. Additionally, to balance the disparities in households' abilities to withstand financial shocks across different economic strata, household economic factors like house ownership, gross agricultural income, gross non-agricultural income, and net worth are also considered [8]. These control variables are meticulously chosen to encompass and adjust for confounding factors influencing liquidity, contingency capacity, and indebtedness, thereby facilitating a more accurate assessment of digital financial inclusion's impact on household financial vulnerability.

Mediating variables in this paper include financial literacy, income from household financial assets, and the total premiums for commercial insurance.

Given the nature of the explained variables as ordered discrete data, the Ordered-Probit model is employed, as delineated in Equations (1), (2).

(1) $${{HFV}_{i}}^{*}=\alpha_{0}+\alpha_{1}\times {DIF}_{i}+\alpha_{2}\times {Control}_{i}+\eta_{i}$$

(2) $${HFV}_{i}={Liu}_{i}+{Emer}_{i}+{Debt}_{i}=\left\{ \begin{array}{c} 0，{{HFV}_{i}}^{*}\leq \gamma_{0}\\ 1，\gamma_{0}\leq {{HFV}_{i}}^{*}\leq \gamma_{1}\\ 2，\gamma_{1}\leq {{HFV}_{i}}^{*}\leq \gamma_{2}\\ 3，\gamma_{1}\leq {{HFV}_{i}}^{*}\leq \gamma_{3} \end{array}\right.$$

In this research, Equation (1) introduces ${DIF}_{i}$ as the core explanatory variable. This represents the digital financial inclusion index for the area where the household is situated, lagged by one period. The variable ${Control}_{i}$ encapsulates a set of essential control variables, inclusive of factors pertaining to household characteristics and household economic conditions.

In equation (2) ， ${HFV}_{i}$ denotes the explained variable, which quantifies the financial vulnerability of each household, assigned values within the 0–3 range. The ${{HFV}_{i}}^{*}$ corresponds to a continuous latent variable representing ${HFV}_{i}$. The parameters $\gamma_{0}$ , $\gamma_{1}$ , $\gamma_{2}$ , $\gamma_{3}$ are the distinct thresholds for ${{HFV}_{i}}^{*}$. The variables ${Liu}_{i}$ , ${Emer}_{i}$ , and ${Debt}_{i}$ respectively represent the liquidity level, contingency capacity, and indebtedness of each household, each being binary variables ranging from 0 to 1.

To empirically test H2, H3, H4, which postulate the mediating effects of digital financial inclusion on household financial vulnerability, this paper employs a mediation effect model as outlined in Equations (3), (4). These equations are structured as follows:

(3) $${Lit}_{i}/{Inc}_{i}/{Pre}_{i}=\varepsilon_{0}+\varepsilon_{1}\times {DIF}_{i}+\varepsilon_{2}\times {Control}_{i}+\eta_{i}$$

(4) $${HFV}_{i}=\beta_{0}+\beta_{1}\times {DIF}_{i}+\beta_{2}\times {Lit}_{i}/{Inc}_{i}/{Pre}_{i}+\beta_{3}\times {Control}_{i}+\eta_{i}$$

In these equations, the mediating variables ${Lit}_{i}$, ${Inc}_{i}$ and ${Pre}_{i}$ represent for each household's financial literacy, income from financial assets, and total commercial insurance premiums, respectively. These mediating factors are integral to understanding the nuanced pathways through which digital financial inclusion influences the financial vulnerability of households.

### 3.3 Descriptive statistics analysis

In this study, provincial-level data were meticulously matched and merged. Following the exclusion of outliers and missing values, the final sample encompassed 27080 households. A notable finding from the dataset is that only 24.09 % of sampled households exhibit no financial vulnerability. Besides, the highest proportion of households, constituting 46.53 %, falls within a financial vulnerability rating of 1.

Table 1 presents the descriptive statistics of the variables. It is observed that the mean value of the household financial vulnerability index is 1.1032. The average values for liquidity level, emergency capacity, and degree of indebtedness stand at 0.3226, 0.6966, and 0.0840, respectively. This suggests that a significant segment of the households faces challenges regarding insufficient emergency savings, while the debt ratio for the majority does not surpass the 30 % threshold. Analysis of the explanatory variables reveals a standard deviation of 0.0866 for the digital financial inclusion index, with the depth of use sub-indicator exhibiting a standard deviation of 0.1340. It reflects that the difference of digital inclusive finance index among provinces is mainly due to the depth of use.

**Table 1:** Descriptive statistics.

Variable Name	Variable Symbol	Obs	Mean	Std.Dev	Max	Min
Household financial vulnerability	HFV	27080	1.1032	0.8031	3	0
Liquidity level	Liq	27080	0.3226	0.4675	1	0
Emergency response capacity	Emer	27080	0.6966	0.4597	1	0
Degree of indebtedness	Debt	27080	0.0840	0.2774	1	0
Digital Inclusive Finance Index	DIF	27080	5.7101	0.0866	5.9342	5.5726
Breadth of coverage	Brea	27080	5.6440	0.0839	5.8689	5.5282
Depth of use	Dep	27080	5.6698	0.1340	5.9925	5.4173
Degree of digitization	Digi	27080	5.9556	0.0487	6.0874	5.8572
Nature of household registration	Reg	27080	0.4164	0.4930	1	0
Household size	Size	27080	3.2940	1.5651	15	1
Age of head of household	Age1	27080	53.8264	12.7462	100	17
Square of the age of head of household	Age2	27080	30.5974	13.7005	100	2.8900
Health status of the head of household	Heal	27080	2.7389	1.0064	5	1
Educational attainment of the head of household	Edu	27080	3.2574	1.5435	9	1
Sex of head of household	Gen	27080	0.2054	0.4040	1	0
Marital status	Mari	27080	2.3159	1.0919	6	1
Family social capital	Cap	27080	0.3198	0.6286	6	0
Child dependency ratio	Chil	27080	0.1243	0.1735	0.8333	0
Old-age dependency ratio	Old	27080	0.3242	0.3961	1	0
Real estate	Prop	27080	0.5951	0.3161	0.9999	0
Gross household income from agriculture	Farm	27080	2.8391	5.0755	15.8754	−15.5010
Gross non-agricultural household income	Nfar	27080	9.8803	3.0728	16.3106	−14.4781
Household net worth	Asse	27080	12.009	4.3028	21.4600	−17.0623
Financial literacy	Lit	27080	0	1	4.0876	−0.7282
Income from household financial assets	Inc	27080	0.1579	2.2971	14.5087	−14.1156
Total commercial premiums	Pre	27080	0.6028	1.8227	13.8158	0

**Note:** Results from Stata 17.0.

In terms of control variables, the mean value for the nature of household registration is 0.4164, indicating a higher representation of non-agricultural households. The gender of the household head has a mean value of 0.2054, signifying a male predominance in the sample's household heads. Additionally, the mean value for household social capital is 0.3198, highlighting that a minority of households have members employed in government institutions or organizations.

For the mediating variables, the mean value of financial literacy among the heads of resident families is 0.0000, signaling an overarching need for enhancement in residents' financial literacy. The mean value for household income from financial assets is 0.1579, with a broad range indicated by the minimum and maximum values, suggesting substantial disparities in investment income across households. The mean value for total commercial insurance premiums stands at 0.6028, reaching a maximum of 13.8158, which indicates that purchasing commercial insurance is not yet a widespread practice among residents.

## 4 Analysis of the empirical results

### 4.1 Baseline regression and analysis

Table 2 illustrates the Ordered-Probit regression outcomes, examining the effect of digital financial inclusion on household financial vulnerability. The regression results are segmented into three categories: without control variables, with control variables pertaining to household characteristics, and with the inclusion of all control variables. A consistent finding across these categories is the significant negative association of the Digital Financial Inclusion Index with household financial vulnerability. This robustness in results, even after the inclusion of various control variables, underscores the efficacy of digital financial inclusion in mitigating household financial vulnerability. Specifically, the regression coefficient of the explanatory variables stands at −1.1157 post the inclusion of all control variables. This implies that a unit increase in the digital financial inclusion index corresponds to a reduction of 1.1157 in household financial vulnerability, thereby affirmatively validating Hypothesis 1.

**Table 2:** Benchmark regression.

Variable	(1)	(2)	(3)
DIF	−1.8618***	−1.2900***	−1.1157***
	(-24.90)	(-16.56)	(-13.86)
Reg		0.1030***	0.0993***
		(6.94)	(6.29)
Size		0.0415***	0.0657***
		(7.49)	(11.48)
Age1		−0.0173***	−0.0148***
		(-4.51)	(-3.82)
Age2		0.0089**	0.0070*
		(2.47)	(1.93)
Heal		0.2060***	0.1625***
		(29.60)	(22.93)
Edu		−0.0771***	−0.0482***
		(-14.15)	(-8.62)
Gen		0.0852***	0.0824***
		(5.04)	(4.82)
Mari		0.0426***	0.0308***
		(6.76)	(4.87)
Cap		−0.0696***	−0.0487***
		(-6.01)	(-4.13)
Chil		0.2764***	0.1828***
		(5.44)	(3.55)
Old		−0.0407	−0.0581**
		(-1.57)	(-2.23)
Prop			0.5612***
			(25.32)
Farm			−0.0140***
			(-9.75)
Nfar			−0.0417***
			(-17.73)
Asse			−0.0651***
			(-36.04)
R^2^	0.0092	0.0406	0.0765
Obs	27080	27080	27080

**Note:** *, **, *** represent significance levels of 10 %, 5 %, and 1 %; z values are presented in parentheses. Results from Stata 17.0.

Delving into the control variables' regression coefficients reveals nuanced insights. For household characteristic factors, the nature of household registration and household size display significant positive correlations with financial vulnerability, indicating heightened vulnerability in agricultural and larger households. Conversely, a higher education level of the household head inversely correlates with financial vulnerability. Additionally, the gender of the household head plays a pivotal role, with male-headed households typically exhibiting reduced vulnerability. This trend can be attributed to generally higher incomes and more rational financial behavior in male-headed households, coupled with enhanced resilience to financial shocks. The employment status of household members also has a significant impact on financial vulnerability, with a significant regression coefficient of −0.0487 for household social capital, most likely because households with family members working in government institutions have stable incomes and are more successful in obtaining loans from formal banks and borrowing from friends and relatives.

In terms of household economic factors, enhancements in total household agricultural income, non-agricultural income, and net worth significantly diminish financial vulnerability, with net worth exerting the most substantial impact. This suggests that greater household funds bolster the capacity to manage financial risks. However, a caveat exists in the translation of income into savings, considering the influence of indebtedness levels. Thus, the selection of housing assets data in this paper holds greater representativeness. The regression coefficient for real estate is 0.5612, indicating that an increase in household real estate proportionally amplifies financial vulnerability. This could be ascribed to the augmented mortgage burden and fragile asset structure stemming from increased household indebtedness, leading to heightened financial difficulties.

### 4.2 Endogeneity test

To address potential endogeneity issues, this study initially utilizes a one-period lagged Digital Financial Inclusion Index as the explanatory variable. This approach aims to eliminate reverse-causality between the explanatory and explained variables. However, endogeneity may still persist due to the omission of certain crucial variables that are either unaccounted for or challenging to quantify.

Given the strong association between digital financial product coverage, market size, and the geographic and industrial characteristics of a region, this paper identifies Hangzhou City, the leading place of Internet finance innovation and home to leading Internet finance enterprises like Ant Financial Services Group, as a focal point. Consequently, the distance between the provincial capital of each household's location and Hangzhou City center in Zhejiang Province, is selected as an instrumental variable for digital financial inclusion.

The two-stage least squares (2SLS) method is employed to assess the validity of the instrumental variable. The results, presented in columns (1) and (2) of Table 3, indicate a significant negative correlation between the instrumental variable distance and digital financial inclusion in the first stage. The F-statistic value substantially exceeds the empirical threshold of 10, negating the possibility of weak instrumental variables. The p-value of 0.00 in the endogeneity test suggests endogeneity in the core explanatory variables, implying that the model may be subject to endogeneity issues.

**Table 3:** Endogeneity test.

Variable	(1)	(2)	(3)	(4)
	DIF	HFV	DIF	HFV
DIS(IV)	−0.1084***		−0.1084***	
	(-142.58)		(-160.33)	
DIF		−1.3305***		−1.9883***
		(-16.10)		(-16.22)
Control	Controlled	Controlled	Controlled	Controlled
F Value of first stage	20330***			
P Value of Endogeneity test		0.0000		
Ln sig2				−2.7757***
Atanhrho_12				0.0960***
R2		0.7104		
AIC		60155.51		−14688.37
Obs	27080	27080	27080	27080

**Note:** *, **, *** represent significance levels of 10 %, 5 %, and 1 %; t values are presented in parentheses in columns (1), z values are presented in parentheses in other columns. Results from Stata 17.0.

Considering that the explanatory and control variables encompass both discrete and continuous types, the accuracy of the 2SLS estimation warrants further examination. Therefore, this paper employs the conditional mixed process (CMP) for model estimation, with results displayed in columns (3) and (4) of Table 3. In the instrumental variables equation, the regression coefficients of the instrumental variables are significantly negative, meeting the correlation criterion for instrumental variables. The endogeneity test parameter atanhrho_12 is significant at the 1 % level, refuting the hypothesis of digital financial inclusion as an exogenous variable and confirming the presence of endogeneity in the baseline model. The impact coefficient of digital financial inclusion in the main equation is −1.9883, surpassing the −1.1157 coefficient from the baseline regression. This suggests that the baseline model may have underestimated the mitigating effect of digital financial inclusion on household financial vulnerability, possibly overlooking variables related to the influence of leading Internet finance enterprises on regional household financial behaviors. The CMP method effectively resolves the endogeneity problem, and its estimation outcomes surpass those of the baseline model. Hence, subsequent analyses in this research will be grounded on the CMP model.

### 4.3 Robustness tests

To fortify the reliability of the model's estimation results, this research employs three distinct methodologies for robustness testing.

Initially, the data from preceding periods are substituted. Specifically, the 2018 Digital Financial Inclusion Index of China (DFIIC) data and the 2019 China Household Finance Survey (CHFS) data, originally used as explanatory variables, are replaced with their 2017 and 2018 counterparts, respectively. The revised estimations, as presented in column (1) of Table 4, reveal that the coefficient of digital financial inclusion on household financial vulnerability remains significantly negative, affirming its robustness at the 1 % significance level.

**Table 4:** Robustness tests.

Variable	(1)	(2)	(3)	(4)	(5)	(6)	(7)
	HFV	HFV	HFV	HFV	Liqu	Emer	Debt
DIF	−0.6586***				−2.3299***	−1.2097***	−1.0194***
	(-4.37)				(-15.35)	(-7.91)	(-4.75)
Brea		−2.4606***					
		(-16.29)					
Dep			−1.2320***				
			(-16.18)				
Digi				−3.1192***			
				(-16.09)			
Control	Controlled	Controlled	Controlled	Controlled	Controlled	Controlled	Controlled
Atanhrho_12	0.0446***	0.1424***	0.0879***	0.0699***	0.1045***	0.0674***	0.0404**
Obs	25385	27080	27080	27080	27080	27080	27080

**Note:** *, **, *** represent significance levels of 10 %, 5 %, and 1 %; z values are presented in parentheses. Results from Stata 17.0.

Subsequently, the explanatory variables are altered. The digital financial inclusion index is decomposed into its three constituent sub-indicators: breadth of coverage, depth of use, and degree of digitization. The estimations, delineated in columns (2) to (4) of the table, consistently exhibit significantly negative coefficients for each sub-indicator, reinforcing the original findings.

Finally, the explained variables are modified. The research reconceptualizes the three dimensions of household financial vulnerability—liquidity level, contingency capacity, and indebtedness level—and recalculates the estimates. The results, as shown in columns (5) through (7) of the table, align congruently with the initial hypotheses.

Collectively, these robustness tests substantiate the primary conclusion, confirming that the model exhibits strong robustness and that the findings are consistent across varied methodological adjustments.

### 4.4 Mechanism analysis

This study conducts a mechanism analysis to understand how digital financial inclusion influences household financial vulnerability, employing a mediated effects model that identifies three key channels.

#### 4.4.1 Enhancing financial literacy of household heads

The estimated results of the mediation effect model for the financial literacy channel are presented in columns (1) and (2) of Table 5. The coefficient of digital financial inclusion on financial literacy, as shown in column (1), is significantly positive. Concurrently, column (2) reveals a significantly negative coefficient of financial literacy on household financial vulnerability. These findings indicate that digital financial inclusion attenuates household financial vulnerability by bolstering the financial literacy of household heads. Digital inclusive finance, leveraging the Internet platform, disseminates financial theory knowledge, encourages rational investment practices, and heightens awareness of real-time market data and key financial indicators. The enhancement of financial literacy among residents，contributes to alleviating household financial vulnerability, substantiating Hypothesis 2.

**Table 5:** Mechanism analysis.

Variable	(1)	(2)	(3)	(4)	(5)	(6)
	Lit	HFV	Inc	HFV	Pre	HFV
DIF	1.0569***	−1.9111***	0.4751***	−1.9883***	0.4035***	−1.9824***
	(16.72)	(-15.52)	(2.85)	(-16.19)	(3.15)	(-16.13)
Lit		−0.0895***				
		(-11.34)				
Inc				−0.0074**		
				(-2.46)		
Pre						−0.0080**
						(-2.07)
Control	Controlled	Controlled	Controlled	Controlled	Controlled	Controlled
Atanhrho_12		0.0973***		0.0963***		0.0957***
R^2^	0.2645		0.0296		0.0903	
Obs	27080	27080	27080	27080	27080	27080

**Note:** *, **, *** represent significance levels of 10 %, 5 %, and 1 %; t values are presented in parentheses in columns (1), (3) and (5), z values are presented in parentheses in columns (2), (4) and (6). Results from Stata 17.0.

#### 4.4.2 Increasing income from household financial assets

Columns (3) and (4) of Table 5 elucidate the mediation effect model results for the financial asset income channel. The coefficient for digital financial inclusion in column (3) is significantly positive, and the coefficient for household income from financial assets in column (4) is significantly negative. These outcomes suggest that digital financial inclusion impacts household financial vulnerability through the augmentation of income from household financial assets. This process is facilitated by the development of digital inclusive finance, characterized by innovative online trading platforms, a plethora of investment and financial management services, and strategic asset risk advisories. These advancements aid households in judiciously allocating financial assets to generate income, thereby enhancing liquidity and improving the capacity to manage risks, ultimately leading to a reduction in financial vulnerability. Thus, Hypothesis 3 is corroborated.

#### 4.4.3 Augmentation of total commercial premiums

The analytical results for the commercial insurance channel are outlined in columns (5) and (6) of Table 5, utilizing the intermediation effect model. The findings demonstrate a significant positive correlation at the 1 % level between digital financial inclusion and total commercial insurance premiums. Simultaneously, total commercial premiums exhibit a significant negative association with household financial vulnerability, also at the 1 % level. This evidences the mediating role of total commercial premiums as an intermediary variable. Digital inclusive finance stimulates residents' interest in commercial insurance investments by facilitating online purchases of insurance products. The presence of insurance assets offers tangible protection to households facing risky shocks, thereby attenuating their economic losses and diminishing their financial vulnerability. Consequently, Hypothesis 4 is validated.

The comprehensive mechanism analysis confirms the efficacy of all three intermediation channels, with financial literacy emerging as the most potent factor in curbing household financial vulnerability. This nuanced understanding underscores the multifaceted impact of digital financial inclusion in enhancing household financial resilience.

### 4.5 Heterogeneity analysis

This study embarks on a heterogeneity analysis to discern the differential impacts of digital financial inclusion across diverse household segments. Reference to the classification of [32], this study discusses heterogeneity in terms of household wealth and location. Therefore, the focus is on the dichotomy of urban versus rural households and the disparity between high-income and low-income households. Here, income level is demarcated based on the median of total household income. The resultant estimations are presented in Table 6.

**Table 6:** Heterogeneity analysis.

Variable	(1)	(2)	(3)	(4)
	Urban	Rural	High-income	Low-income
HFV	1.0297	1.2063	0.9280	1.2780
Farm + Nfar	10.4725	9.4055	11.6120	8.4445
DIF	−1.9606***	−2.0295***	−1.4486***	−2.1548***
	(-12.78)	(-9.90)	(-8.86)	(-10.85)
Atanhrho_12	0.0991***	0.0945***	0.0807***	0.1015***
Obs	15804	11276	13540	13540

**Note:** *, **, *** represent significance levels of 10 %, 5 %, and 1 %; z values are presented in parentheses. Results from Stata 17.0.

Columns (1) and (2) of Table 6 provide insights into the heterogeneity based on the urban-rural gap. The mean value of household financial vulnerability for urban households is 1.0297, compared to 1.2063 for rural households, signifying greater financial stability and comprehensive risk mitigation measures in urban settings. When examining the estimated coefficients of digital financial inclusion on household financial vulnerability, the analysis reveals a more pronounced impact on rural households. The mitigating effect on their financial vulnerability is notably stronger. This differential may be attributed to the limited presence and scope of financial services in rural areas, where residents often encounter higher costs and time expenditure in accessing financial services. This situation may compel rural households towards riskier financial behaviors, such as private lending. Furthermore, rural households often grapple with information asymmetry and a lack of clarity regarding various financial products and services, impeding their ability to make informed investment and financial management decisions. The advent of digital inclusive finance has ameliorated these challenges by providing rural households with more streamlined and accessible financial services, thereby significantly transforming their investment and financial management behaviors. This has enhanced their financial literacy, increased their asset incomes, and provided additional protective measures, collectively reducing their financial vulnerability.

Columns (3) and (4) of Table 6 delve into the income-level heterogeneity. The analysis reveals a stark contrast in the financial vulnerability between high-income and low-income households, with the latter experiencing more severe financial vulnerability issues. The sensitivity of low-income households to changes in digital financial inclusion is more pronounced, likely due to their smaller income-expenditure balances, lower savings levels, and thus weaker liquidity and contingency capacities. Digital financial inclusion has endowed these households with a broader spectrum of investment and financial products and services. It has facilitated access to funding sources for households lacking sufficient collateral and cash flow, directly uplifting household income levels and amplifying the influence of digital financial inclusion on the financial vulnerability of low-income households.

## 5 Conclusions and recommendations

### 5.1 Principal conclusions

This research utilizes the 2019 China Household Finance Survey (CHFS) data at the provincial household level and the 2018 Digital Financial Inclusion Index of China (DFIIC) to examine the influence of digital financial inclusion on household financial vulnerability. Employing the Ordered-Probit model, the study addresses endogeneity through the instrumental variable method, substantiates its findings via robustness tests, analyzes impact mechanisms using mediation effects, and conducts heterogeneity analysis based on household and income classifications. The study arrives at the following conclusions:

The benchmark regression results robustly indicate that digital inclusive finance significantly reduces the financial vulnerability of households. Examining household characteristic factors, it emerges that agricultural and larger households are more susceptible to financial vulnerability. Conversely, households headed by males with better health and higher education levels exhibit greater efficacy in circumventing financial risks. Interestingly, a higher proportion of elderly individuals in households does not exacerbate financial vulnerability but rather seems to diminish associated risks.

In terms of household economic factors, the analysis reveals that an increase in household net worth and total income effectively prevents risk and reduces financial vulnerability. However, it is notable that households with a substantial proportion of real estate assets experience heightened financial vulnerability. This is attributable to the fact that in many households, increased house ownership is often linked with rising indebtedness levels, thereby escalating financial risk. Post endogeneity correction and robustness verification, it is observed that for every unit increase in digital financial inclusion, there is a corresponding reduction of 1.9883 in household financial vulnerability.

Mechanism analysis highlights that digital inclusive finance can alleviate household financial vulnerability through three pathways: enhancing the financial literacy of household heads, augmenting household financial asset income, and increasing commercial insurance purchases. Of these, improving financial literacy is identified as the most impactful channel. Furthermore, the heterogeneity analysis reveals that rural households and those with lower income levels are more dependent on digital inclusive finance. The greater the advancement in digital financial inclusion, the lower the financial vulnerability observed in such households.

### 5.2 Policy recommendations

In light of the findings and considering the current dynamics of China's financial markets and household financial vulnerability, this paper proposes the following policy recommendations:

#### 5.2.1 Enhancing digital financial inclusion

Given that digital inclusive finance markedly alleviates household financial vulnerability, particularly for agricultural households and low-income groups, it is imperative to accelerate its development across all regions. In rural and remote areas, it is crucial to enhance the financial service infrastructure. This entails improving digital service infrastructures, such as mobile networks, lowering regional network tariffs, and expanding the accessibility of digitally inclusive financial services. Such initiatives aim to enable a broader range of households to access the benefits of digital financial inclusion.

#### 5.2.2 Elevating financial literacy among the population

Disadvantaged groups, particularly in less developed regions, may not fully harness the benefits of mobile internet technology and its financial applications due to limited financial awareness and management skills. Local governments should tailor digital inclusive financial services to their region's specific needs, promoting financial education and enhancing overall financial literacy among residents. This includes disseminating current financial policies, consolidating financial information, cautioning against financial fraud, with a focus on household heads. As household financial management often hinges on the head's decision-making, elevating their financial literacy is fundamental to fundamentally mitigating household financial vulnerability.

#### 5.2.3 Accurately understanding household needs

Financial institutions should leverage digital technology to create user-friendly, intuitive financial service platforms catered to rural and low-income families. This approach involves removing information barriers and minimizing transaction costs, offering a diverse range of financial products and services tailored to user needs. Providing online consultations and specialized advice based on individual family financial circumstances can assist households in adjusting their asset structures and effectively managing financial risks. This strategy, encompassing mechanisms to enhance income from family financial assets and extend commercial insurance protection, facilitates households in fortifying their financial resilience.

## Data availability

CHFS data is freely available for download at https://chfser.swufe.edu.cn/datas, and PKU-DFIIC data can be accessed through https://idf.pku.edu.cn. Data will be made available upon request.

## Ethics statement

All procedures performed in this study were in accordance with the ethical standards of the university. Ethical clearance and approval were granted by Zhejiang University of Finance and Economics. All participants provided informed consent before joining the study.

## CRediT authorship contribution statement

**Jianhe Liu:** Writing – review & editing, Writing – original draft, Visualization, Validation, Supervision, Software, Resources, Project administration, Methodology, Investigation, Formal analysis, Data curation, Conceptualization. **Yuan Chen:** Writing – review & editing, Writing – original draft, Visualization, Validation, Supervision, Project administration, Investigation, Formal analysis, Data curation, Conceptualization. **Xuanyu Chen:** Writing – review & editing, Writing – original draft, Visualization, Software, Resources, Project administration, Investigation, Formal analysis, Data curation, Conceptualization. **Bin Chen:** Writing – original draft, Visualization, Software, Resources, Project administration, Investigation, Formal analysis, Data curation, Conceptualization.

## Declaration of competing interest

The authors declare that they have no known competing financial interests or personal relationships that could have appeared to influence the work reported in this paper.
